# Timing and rate of spheno-occipital synchondrosis closure and its relationship to puberty

**DOI:** 10.1371/journal.pone.0183305

**Published:** 2017-08-21

**Authors:** Anwar Alhazmi, Eduardo Vargas, J. Martin Palomo, Mark Hans, Bruce Latimer, Scott Simpson

**Affiliations:** 1 Department of Orthodontics, School of Dental Medicine, Case Western Reserve University, Cleveland, Ohio, United States of America; 2 Department of Biology, School of Art and Science, Case Western Reserve University, Cleveland, Ohio, United States of America; 3 Department of Anatomy, School of Medicine, Case Western Reserve University, Cleveland, Ohio, United States of America; Medical University of South Carolina, UNITED STATES

## Abstract

**Objectives:**

This study examines the relationship between spheno-occipital synchondrosis (SOS) closure and puberty onset in a modern American population. It also investigates the timing and the rate of SOS closure in males and females.

**Materials and methods:**

The sample includes cross-sectional and longitudinal 3D Cone Beam Computed Tomography (CBCT) scans of 741 individuals (361 males and 380 females) aged 6–20 years. Each CBCT scan is visualized in the mid-sagittal plane, and the spheno-occipital synchondrosis (SOS) is scored as completely open, partially fused, mostly fused, and completely fused. The Menarche commencement is used as an indicator of puberty onset in females.

**Results:**

Mean ages of open, partially-fused, mostly-fused, and completely fused SOS were 11.07, 12.95, 14.44, and 16.41 years in males, and 9.75, 11.67, 13.25, and 15.25 in females, respectively. The results show there is a significant association between the SOS closure stage and the commencement of menarche (Fisher's Exact Test p < 0.001). It was found that females had a higher SOS closure rate (38.60%) per year than males at the age of 10 years. The closure rate in males appears slower than females at age 10, but it lasts a longer time, ranging between 22 and 26% per year from age 11 to 14 years.

**Conclusion:**

There is a significant relationship between puberty onset and SOS closure, suggesting its closure is at least partially affected by systemic, hormonal changes in the growing adolescent. Also, SOS closure occurs at a faster rate and at an earlier age in females compared to males.

## Introduction

The spheno-occipital synchondrosis (SOS) is a cartilaginous growth center between the occipital and sphenoid bones. It is composed of hyaline cartilage, which is abundant during the growth phase of the cranial base and then ossifies during skeletal maturation [1]. Orthodontics and craniofacial growth studies show that SOS plays a vital role in cranial base growth, which participate in defining the final shape of the cranial base and its relation with the upper and lower jaws [2–4]. A recent study found a strong correlation between premature closure of the SOS and mid-face hypoplasia [5]. The study of SOS development and its role in craniofacial growth is an important area of research in craniofacial growth. In addition, much research has focused on using the spheno-occipital synchondrosis development for age estimation. According to Shirley and Jantz [6], the progress of spheno-occipital synchondrosis closure can help to provide a means to estimate age in adolescents. Knowing the accurate age of SOS closure can have significant applications in the medical, forensic, and anthropological fields [7].

The age of SOS ossification is relatively late, compared with other cranial base synchondroses that fuse prenatally (inter-sphenoid) or during early childhood (spheno-ethmoidal) [8,9]. There is still considerable disagreement in literature about when the spheno-occipital synchondrosis fuses. The varied reports on closure age between studies could be due to using different methods for SOS closure analysis by different researchers [10]. These methods include direct inspection of skeletal material, autopsy, bone histology, conventional radiography, and recently, computed tomography 3D imaging. Early studies assert complete closure between 17 and 25 years in males and females [8,11]. Later, differences in closure age between males and females were reported with females beginning to fuse between 11 and 14 years and males between 13 and 16 years [12–18]. More recently, Computed Tomography (CT) and Cone Beam Computed Tomography (CBCT) have been used in SOS evaluation. Due to superior visualization achieved using 3D radiographic scans, more recent studies have used it to evaluate the skull base, claiming it could result in more accuracy in determining SOS closure [15,19]. Recent studies using CT imaging reported age of closure would start around 11 years of age in female and around 13 years in males [15,17,18]. However, other studies that used CT scans reported closure could start as early as 4 years [4,20–22].

Clearly, most SOS age estimation research has been limited to studying the SOS closure from an anatomical point of view, without considering the broader systemic physiological changes that could be associated with SOS closure. The majority of earlier research was done using cadavers or skeletal material, which makes it difficult to answer clinical questions that require living individuals [6,14,17,22–24].

One question that must be answered is whether there is a relationship between systemic growth changes during adolescence and closure of the SOS. As Scheuer and Black stated [16], “the closure of spheno-occipital synchondroses almost certainly occurs during adolescence rather than the young adult period” (2004:77) and they add, “it appears that the fusion times of both the intra-occipital and the spheno-occipital synchondroses are related to significant maturational events” (2004:77). However, to the authors’ knowledge, no study examined this claim and clarification of the relationship between SOS closure and puberty changes. This relationship, if it exists, could help to explain some of the variability of the age of SOS closure between different populations and studies, allowing a better understanding of the basic biology of SOS development, and it will help to narrow the expected age of SOS closure.

This research used cross-sectional and longitudinal data from a sample of modern Americans aged 6–20 years old who had standard orthodontic treatment at the Case Western Reserve University (CWRU) School of Dental Medicine Graduate Clinic and had 3D Cone Beam Computed Tomography (CBCT) scans during their treatment. These high-resolution 3D images will help to describe accurately and better understand the timing and nature of the SOS closure process. The advantage of using longitudinal data is that, we can evaluate the rate of SOS closure in individual males and females. Finally, since our sample includes active patients in the CWRU-SODM Orthodontics clinic, we reviewed female patients’ medical charts and explored the relationship between puberty and SOS closure in females.

The objectives of this study were: (1) to document the timing and pattern of SOS closure in a large modern sample of known-age males and females; (2) to examine the correlation between SOS closure and puberty onset in females; and (3) to calculate the rate of SOS closure per year.

## Materials and methods

### Sample

**Fig 1:**
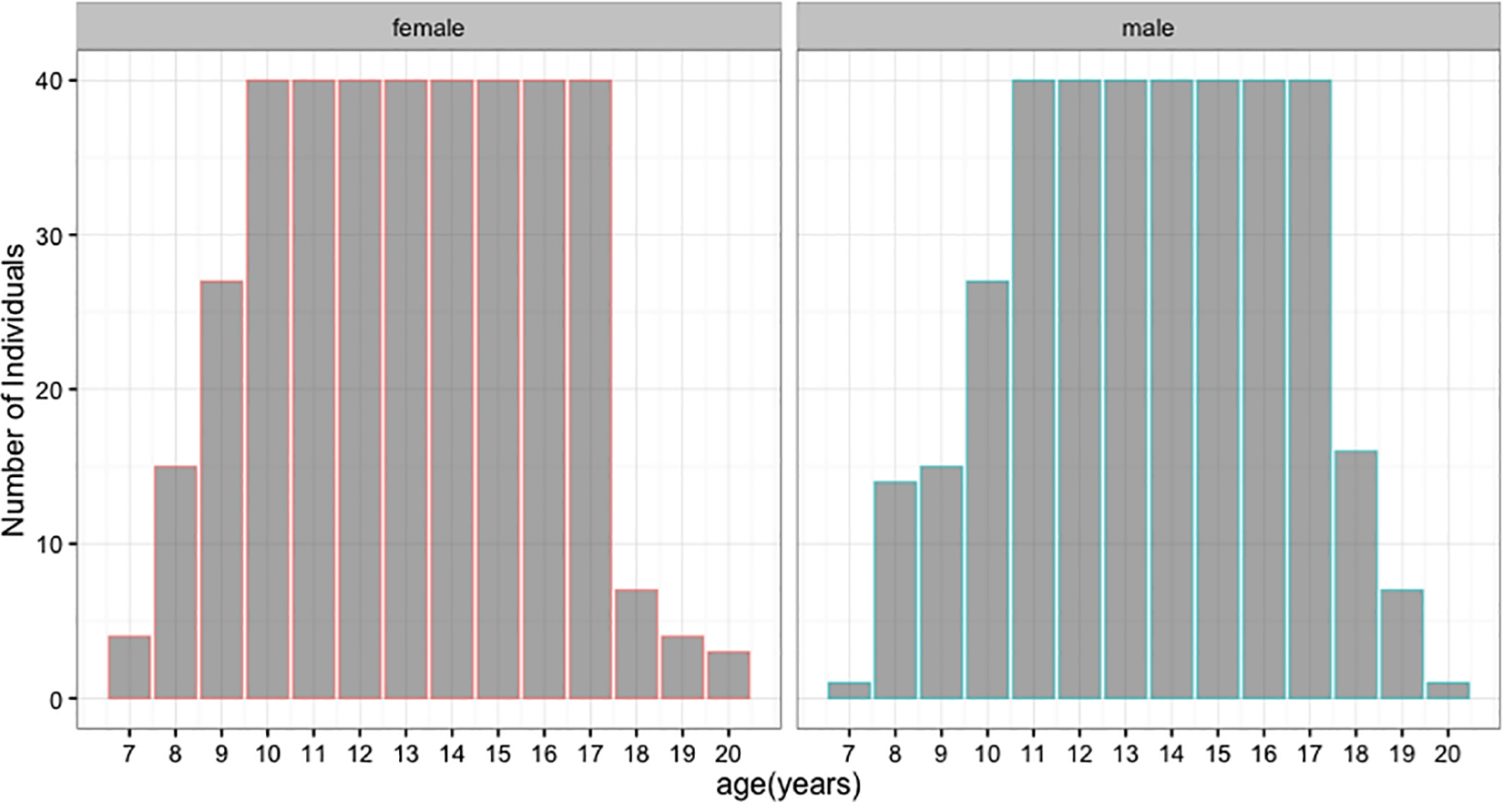
Age and sex distribution of the study sample.

This study examines high resolution Cone Beam Computed Tomography (CBCT) scans of 741 individuals (361 males and 380 females) with a mean age of 13.30 ± 2.85 years (range = 6.7–20.4 years) (Fig 1). The study sample was randomly selected from the digital patient database of the Department of Orthodontics, Case Western Reserve University (CWRU) School of Dental Medicine, Cleveland, Ohio. Pre-existing records between 2009 and 2016, including clinical examination, CBCT scans, and medical history charts of a sample of patients, were used. The database was stratified to subgroups according to age and sex. Most patients in the database were between ages 10 and 17 years, and we randomly selected 40 individuals per age group per sex for the analysis. For individuals younger than 11 years and older than 17 years, we included all available records that met the inclusion criteria. The inclusion criteria include records that have clear CBCT scans without distortion or abnormal morphologies resulting from pathology, trauma, and developmental and/or congenital disorders. All scans were taken in the Craniofacial Imaging Center at the CWRU School of Dental Medicine, using the CBCT machine (CB MercuRayTM, Hitachi Medical Systems America Co.–Twinsburg, OH). The scanner was customized for CWRU with the following specifications: 2 mA, 120 kVp, 12-inch field of view (F), 512 slices, 0.377 mm slice thickness, 1024x1024 pixels resolution, 12 bits per pixel, 4096 gray scale [25,26]. The volumetric data of scans were imported in DICOM format into Dolphin 3D (version 11, Dolphin Imaging and Management Systems, Chatsworth, CA). The 3D reconstruction was visualized on a 21.5-in screen monitor with a display resolution of 1920X1080 pixels. Demographic and scanning data, like the date of birth, date of scans, sex, and age, were recorded on a separate sheet. The CWRU Institutional Review Board approved the study protocol used in this retrospective study (IRB-2016-1422). All patients used in this study were obtained from the patient database of the Department of Orthodontics, Case Western Reserve University. Each patient had a signed consent form, allowing the use of orthodontic records.

### Imaging and scoring

**Fig 2:**
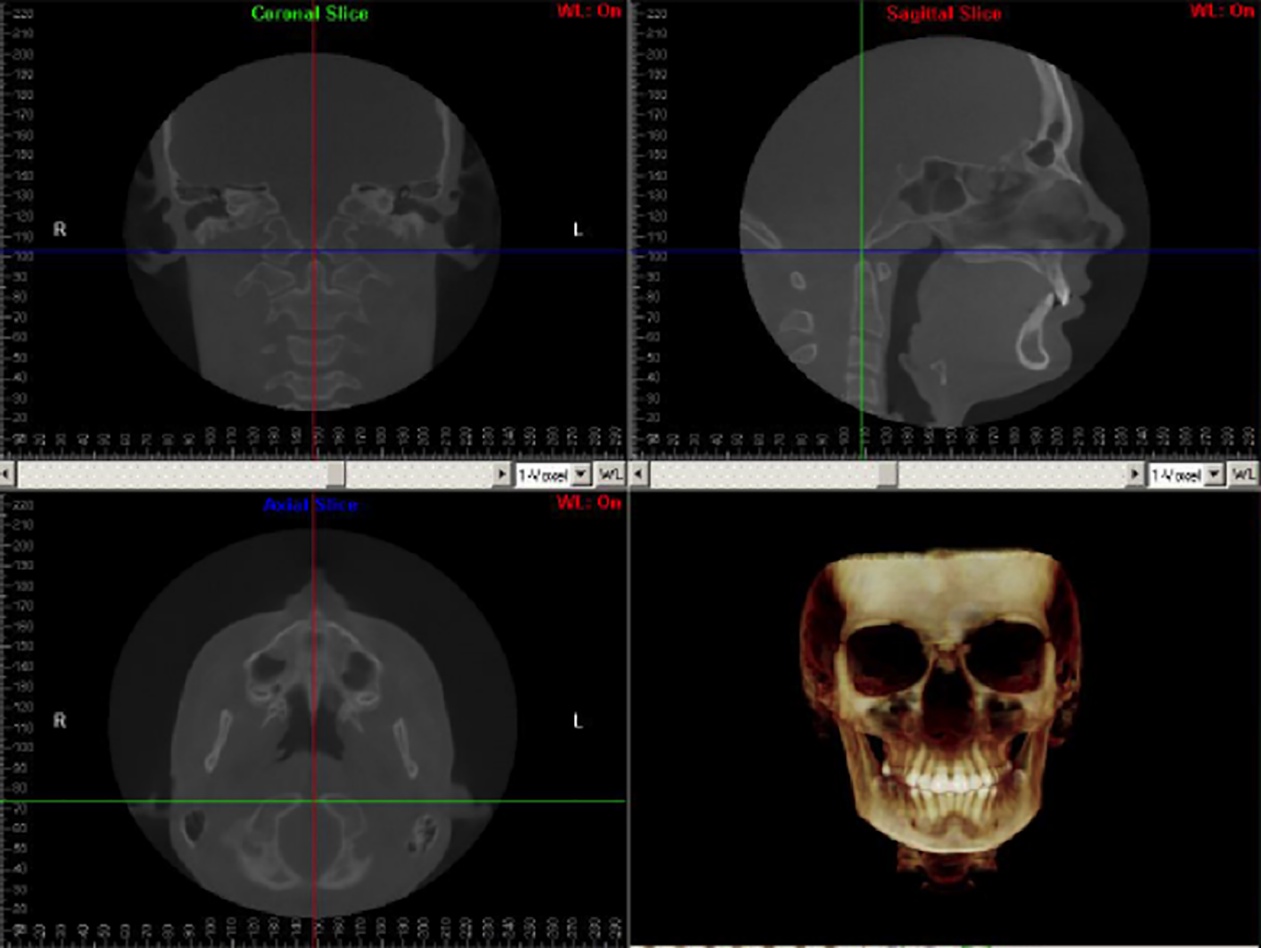
Mid-sagittal CBCT evaluation of spheno-occipital synchondrosis while the head in the default orientation.

**Fig 3:**
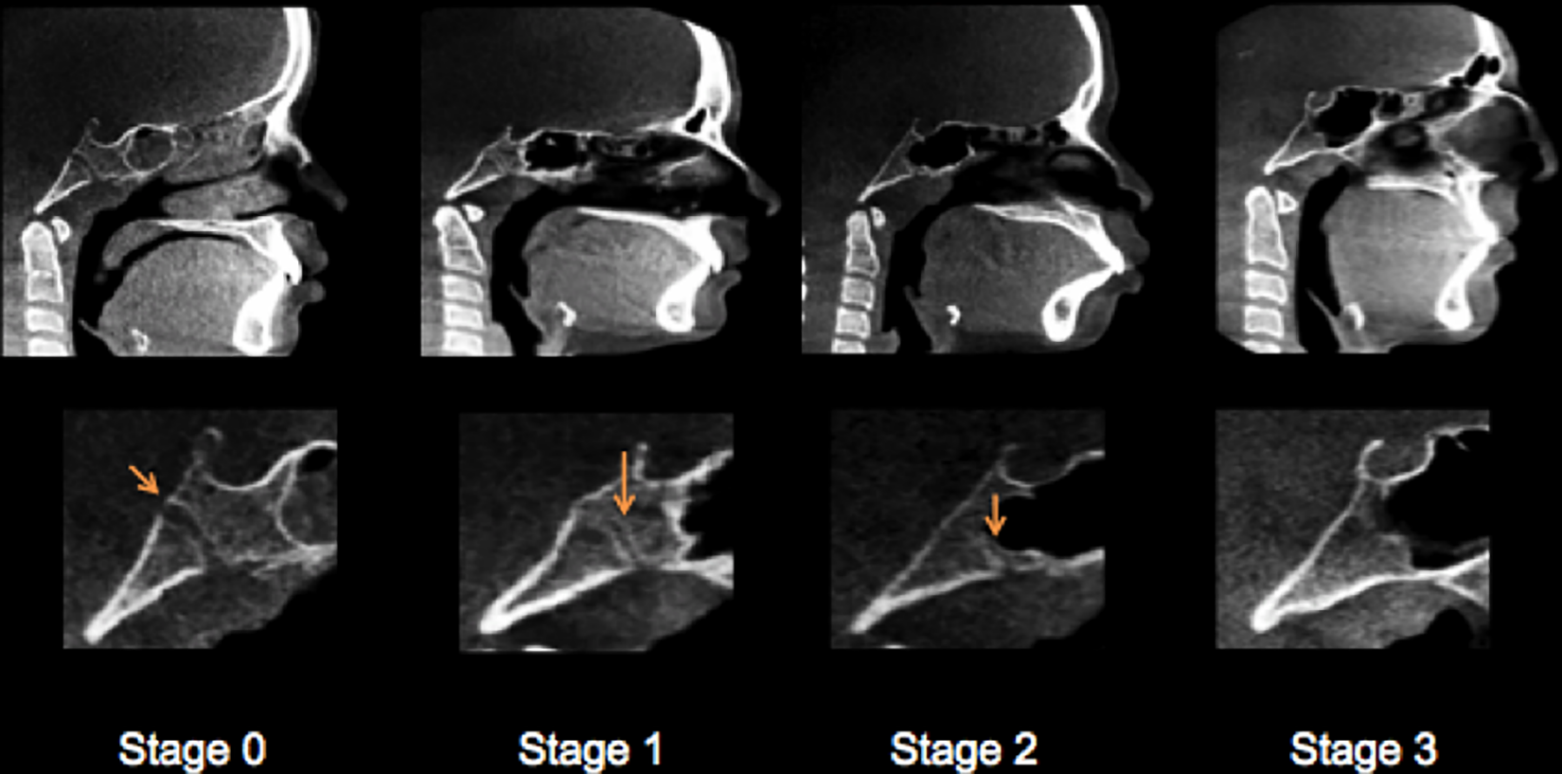
Mid-sagittal CBCT images demonstrating the spheno-occipital synchondrosis closure Stages. Stage 0, unfused. Stage 1, fusing endocranially. Stage 2, fusing ectocranially. Stage 3, complete fusion.

Patients’ scanning was conducted while they were sitting upright with a natural head position (NHP), looking at a remote point at their eye level. A 3D virtual model was created using the Dolphin software program for each individual and used to establish head orientation and standardize the center of the 3D coordinate system. Orientation of the 3D model was done following a standardized method used at CWRU; description is given in S1 Protocol. Visualization of the synchondrosis ossification was done in mid-sagittal view while setting in default orientation (Fig 2). The SOS fusion was calculated as a percentage of a straight line that passes through the center of SOS from ectocranial to the endocranial sides. Two observers, one orthodontist (A.A.) and one dentist (E.V.), were trained together to ensure observational consistency for scoring SOS on a test sample of 30 images not included in the final analysis. Each observer evaluated the SOS status in mid-sagittal view. The slice thickness was 1 mm. After the calibration process, each observer independently scored the whole test sample according to a four-stage system [17]. The synchondrosis was scored as complete open (Stage 0, Unfused), partial closure (Stage 1, Fusing endocranially, ≤ 50%), mostly fused (Stage 2, Fusing ectocranially, > 50% and less than 100% closure), and completely fused (Stage 3) (Table 1 and Fig 3). After finishing scoring, another session was held to reach a consensus between the observers on cases of disagreement. In this Stage, additional axial images were used for assessment in addition to the mid-sagittal one following Okamoto et al. method [19]. Individual ages and scores of spheno-occipital synchondrosis are given in S1 and S2 Tables.

**Table 1:** Definition of the staging system used for scoring spheno-occipital synchondrosis closure degree.

Stage		Description
0	Unfused	Completely open with no evidence of fusion between the basilar portion of the occipital and the sphenoid, no bone present in the gap.
1	Fusing endocranially	No more than half the length of the synchondrosis is fused proceeding endo- to ectocranially.
2	Fusing ectocranially	Greater than half the length of the synchondrosis is fused, the ectocranial (inferior) border remains unfused.
3	Complete fusion	Completely fused with the appearance of normal bone throughout, a fusion scar may be present.

### Statistical analysis

Statistical analysis was performed using R software (R Core Team 2015—R: A language and environment for statistical computing. R Foundation for Statistical Computing, Vienna, Austria. URL https://www.R-project.org/). Descriptive statistics were calculated for both genders to determine the sample distribution, the means, and the standard deviations of the mean ages for the SOS Stages. Spearman correlation analysis was used to assess the correlation between age and the closure stage. The difference between stages and gender according to age and the comparison between gender and age considering stages were determined by Mann Whitney/Wilcoxon Two-sample test and Kruskal-Wallis test for 4 closure stage groups. Regression analyses were conducted using age as a dependent variable and the stage of spheno-occipital closure as an independent variable for each gender. Prediction of average ages for each closure stage for both genders were calculated based on linear regression parameters. Intra- and inter-observer errors were quantified using Cohen’s Kappa coefficient, calculated using the scores assigned for the total sample. Intra-observer error was calculated based on the repeated assessment of 50 images, with a 1-month interval between the first and second scoring sessions. Fisher’s exact test was used to evaluate the relationship between SOS closure stage and commencement of menarche. For all analyses, P < 0.01 was considered statistically significant.

## Results

Based on the repeated assessment of 50 CBCTs for 50 individuals, observer 1 (A.A.) had 3/50 non-agreements and observer 2 (E.V.) had 5/50 non-agreements (Table 2). The Weighted Kappa measure of agreement for A.A. is 0.94 (P <0.001) and for E.V. is 0.93 (P <0.001); the strength of agreement between repeated observations is rated as “almost perfect” [27]. Inter-observer concordance based on the analysis of the total sample is 0.85 (P<0.001), also considered an almost perfect agreement (Table 2).

**Table 2:** Inter and intra-observer reliability.

	Intraobserver		Interobserver
	A.A.	E.V.	Both
Number of cases	50	50	741
Score of agreement (%)	94%	93%	91%
Weighted Kappa (*k*)	0.92	0.88	0.89
Significance	P < 0.001	P < 0.001	P < 0.001

**Fig 4:**
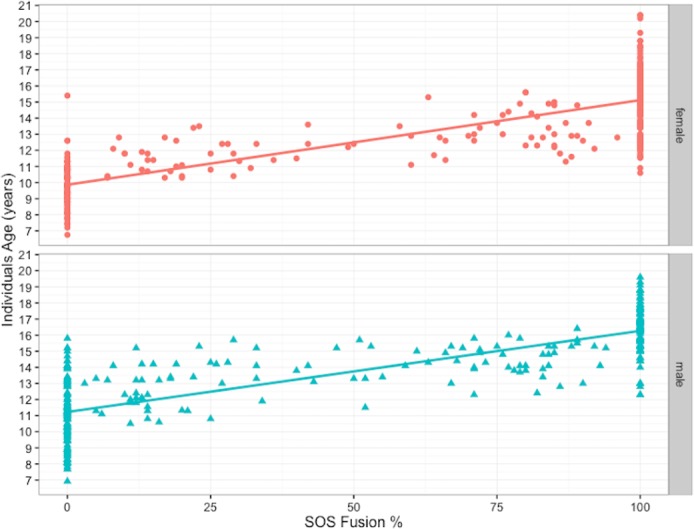
Correlation between age and spheno-occipital synchondrosis closure in males (n = 361) and females (n = 380).

Spearman rank correlations indicate a significant positive relationship between age and stage of spheno- occipital closure for both sexes (male: *r*_*s*_ = 0.844; sig = *P* < 0.001 female: *r*_*s*_ = 0.843; sig = *P* < 0.001) (Fig 4). The youngest age for Stage 3 (complete fusion) attainment is 12.30 years in males (mean 16.41 ± 1.40 years) and 10.66 years in females (mean 15.25 ± 1.89 years). The latest age at which the SOS remains completely open (Stage 0) is 15.80 years (mean 11.07 years ± 1.91) and 15.40 years (mean 9.75 ± 1.30 years) for males and females, respectively (Table 3).

**Table 3:** Descriptive statistics for spheno-occipital synchondrosis closure in study population.

	n	Mean age (years)	SD	95% CI mean age	Median age	Range
Males						
Stage 0	145	11.07	1.91	10.75–11.37	11.08	6.90–15.80
Stage 1	48	12.95	1.38	12.54–13.34	13.20	10.50–15.70
Stage 2	48	14.44	1.06	14.13–14.75	14.60	11.50–16.40
Stage 3	120	16.41	1.40	16.15–16.66	16.50	12.30–19.60
Females						
Stage 0	110	9.75	1.3	9.50–10.00	9.82	6.75–15.4
Stage 1	37	11.67	0.93	11.35–11.97	11.80	10.30–13.60
Stage 2	44	13.25	1.19	12.89–13.61	12.95	11.10–15.60
Stage 3	189	15.25	1.89	14.97–15.51	15.40	10.60–20.40

**Fig 5:**
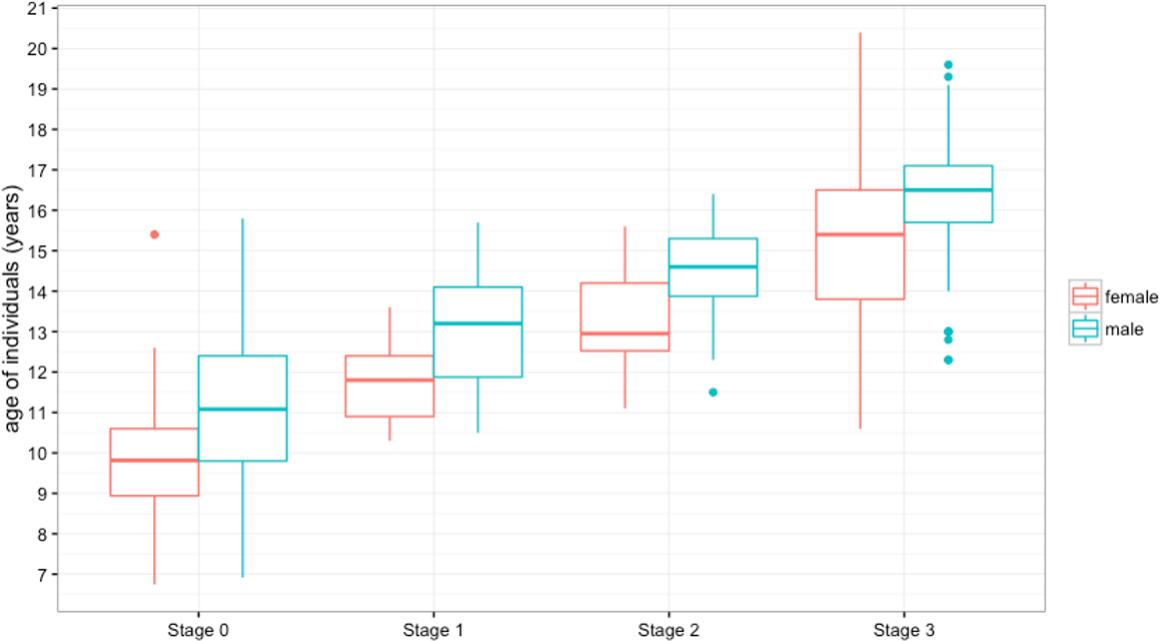
Distribution of study population according to age, sex, and spheno-occipital synchondrosis closure Stage (median and quartile range). See Table 3 for data.

Table 3 shows the distribution and mean ages of all subjects grouped by SOS Stages and gender. Each stage in the female sample appeared earlier than the corresponding stage in males (Fig 5). A Kruskal-Wallis nonparametric test for 4-group comparisons of the mean ranks between groups showed highly significant differences in both genders. The chi-square values were χ^2^ (3) = 272.95; (p <0.001), and χ^2^ (3) = 256.40; (p <0.001) for the females and the males, respectively. The Dunn multiple comparison procedure with the Bonferroni adjustment was used to test for intragroup SOS Stage differences in age for males and females separately and it showed significant age differences between all Stages (p < 0.001).

**Fig 6:**
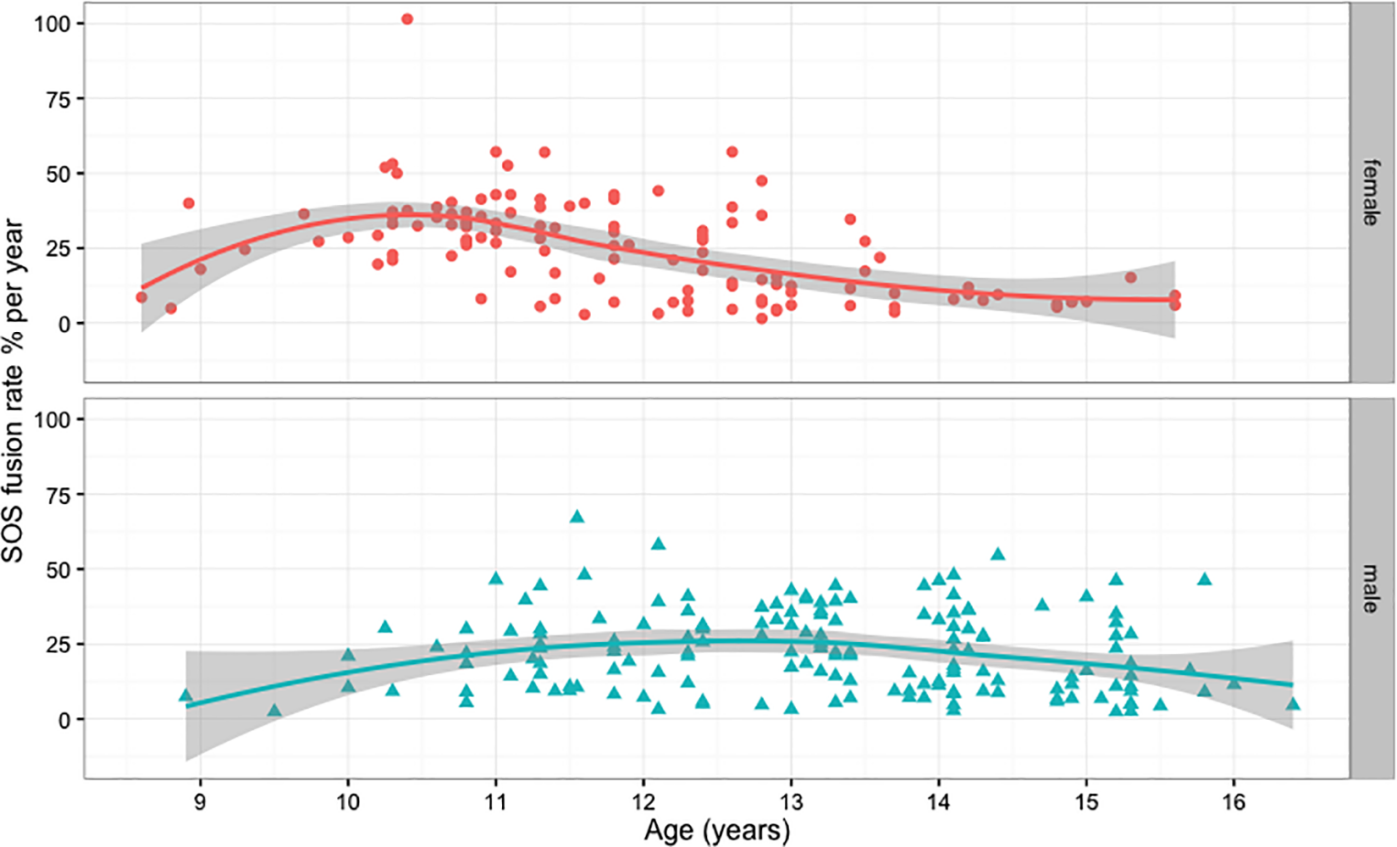
Males and females spheno-occipital synchondrosis closure rate per year (percentage). The Scatterplot with nonlinear fit curve and 95% CI shows the data from Table 7 and Table 8.

Simple least square regression was carried out separately for males and females, comparing SOS closure percentage with corresponding age. The slope of regression line indicated that the age tends to increase as SOS closure percentage increases (Fig 6). When assessing the applicability of the regression model in accounting for age variation (adjusted R2), the variation of spheno-occipital synchondrosis closure percentage explains 66.7% and 68% of the age variation in males and females, respectively (Table 4). To find the effect of each stage of the 4-closure stage groups (categorical predictors) on the age, we included dummy variables in our regression model using only zeros and ones [28]. The results for the regression analysis show the 95% CI parameters for the β coefficients and the constant, which are used to construct the regression equation from which the upper and lower limits of an age estimate are calculated (Table 5). The regression model was expressed by this equation:

$$ϒ=\alpha +\beta_{a}X_{a}+\beta_{b}X_{b}+\beta_{c}X_{c}\pm \varepsilon$$

Where ϒ is the dependent variable (age) and α is the ϒ-intercept for the regression line and represents the baseline or Stage 0, *β* is the slope of the regression line for the independent variable *X* at 3 levels (Stages 1, 2 and 3) and *ε* is the error term used to construct an age range. The equation becomes:

Formales,Age=11.06+1.78(Stage1)+3.87(Stage2)+5.34(Stage3)±ε

ForFemales,Age=9.74+1.91(Stage1)+3.50(Stage2)+5.49(Stage3)±ε

Whenever an individual is assigned to a specific stage, this gives it variable one, and the rest are zeros.

**Table 4:** Simple least square regression output for spheno-occipital synchondrosis closure.

R Software output	Males	Females
F	723.2 (p < 0.001)	808.6 (p < 0.001)
Adjusted R^2^	0.667	0.680
Residual S.E.	1.618	1.624
Pearson (r)	0.816	0.824

**Table 5:** Regression output for spheno-occipital synchondrosis closure in relation to age.

	Coefficient B	Standard Error	95% CI
Males			
Stage 0 (Intercept)	11.06*	0.13	10.80–11.32
Stage 1	1.78*	0.26	1.35–2.39
Stage 2	3.87*	0.26	2.85–3.89
Stage 3	5.34*	0.20	4.95–5.72
Females			
Stage 0 (Intercept)	9.74*	0.15	9.45–10.04
Stage 1	1.91 *	0.30	1.32–2.51
Stage 2	3.50*	0.28	2.94–4.06
Stage 3	5.49*	0.19	5.12–5.87

*P < 0.01 (significant difference between stages within each group)

The regression output presented in (Table 5) were used to produce prediction intervals of age based on SOS stage. Age range prediction intervals based on the regression model has produced for each stage in males and females using +1 and +2 standard deviations for the 68% and 95% prediction intervals, respectively (Table 6). Age limit was derived from the upper bound of age in Stage 0 (open SOS). This age limit gives the oldest age at which an individual is likely to have an open SOS, which is 11.33 years in females and 12.65 years in males. Similarly, the ‘‘fused” age limit was derived from the lower bound of age in Stage 3, and this gives the youngest age at which an individual is likely to have a fused SOS; these age limits were 13.66 and 14.81 years in females and males, respectively. The ‘‘fusing” age ranges are composed of partial closure (Stage 1) and mostly closure (Stage 2) intervals, which were also derived from age bounds using +1 and +2 standard deviations for the 68% and 95% prediction intervals, respectively (Table 6).

**Table 6:** Age ranges for modern individuals based on regression analysis probability distribution (PI, prediction interval).

	Females		Males	
SOS closure stage	68% PI	95% PI	68% PI	95% PI
Stage 0	< 11.33	<12.88	< 12.65	<14.20
Stage 1	10.06–13.26	8.50–14.82	11.34–14.54	9.78–16.10
Stage 2	11.65–14.85	10.10–16.40	12.84–16.04	11.28–17.60
Stage 3	> 13.66	> 12.12	> 14.81	> 13.27

**Fig 7:**
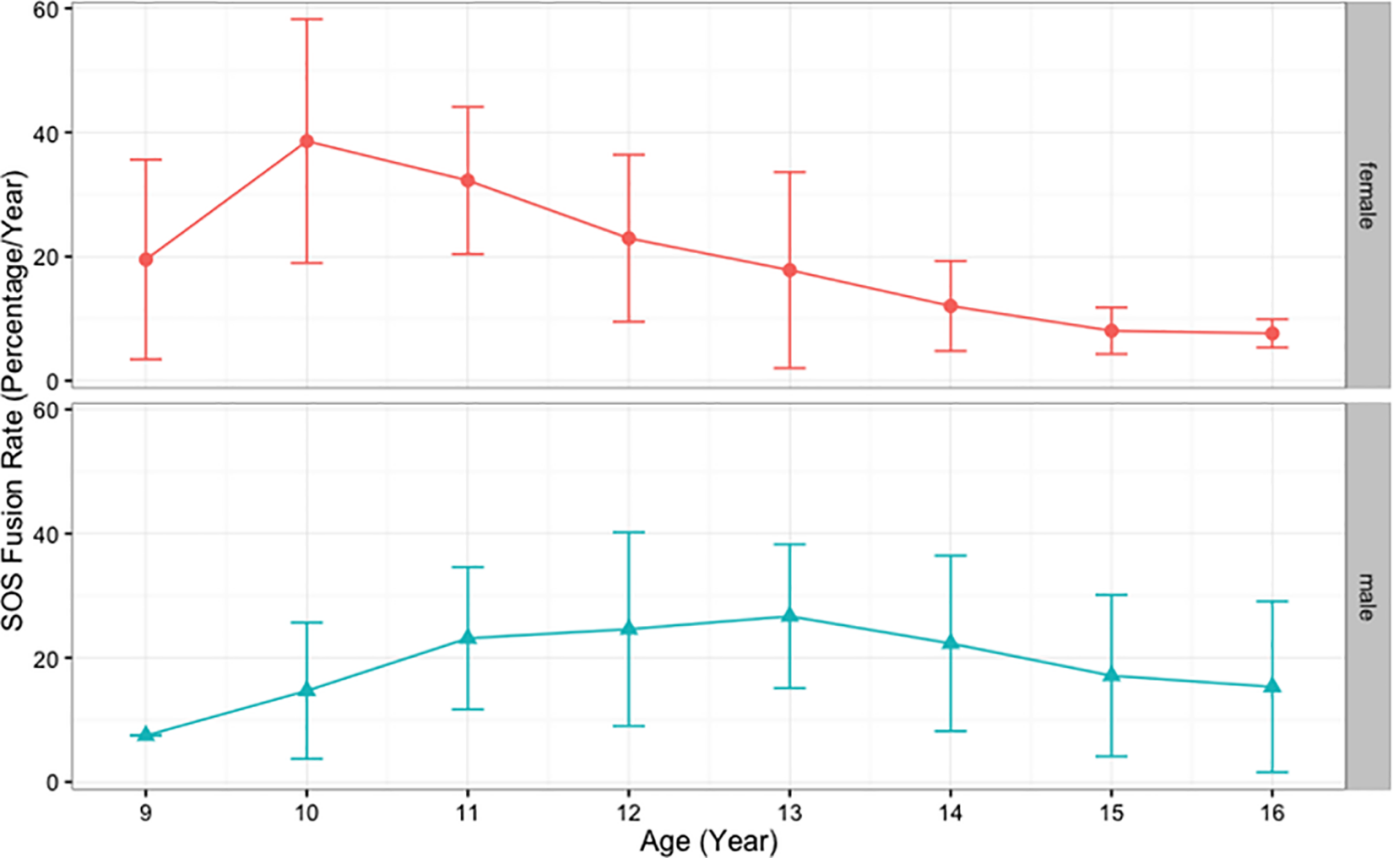
Means and SD of spheno-occipital synchondrosis closure rate per year (percentage) in males and females, see Table 7 and Table 8 for data.

In the analyses described above, we used only 1 CBCT for each patient. For SOS fusion rate analysis, records were reviewed to select individuals who had another CBCT at the end of their treatment. After excluding the cases that had no changes (i.e., cases with T1 at Stage 0 and T2 at Stage 0 or cases with T1 at Stage 3 and T2 at Stage 3), we had 121 females (242 CBCT records) and 159 males (318 CBCT records) with 2 consecutive CBCTs for each individual. Duration between 2 CBCT scans in this sample ranged between 10 and 60 months. The SOS closure percentage differences between T1 and T2 CBCT scans were calculated by subtracting the percentage of SOS fusion at T2 from the percentage of SOS fusion at T1. The SOS closure difference between T1 and T2 were then divided by the duration between T1 and T2 scans in years to calculate the rate of SOS fusion. It is worth mentioning the duration between T1 and T2 does not record exact fusing time; it only records the time between 2 scans and between which we can measure the amount of closure that has occurred. The rate of SOS closure was calculated for both genders. The closure rate was plotted against age at T1, which shows different patterns between males and females (Fig 6). Age groups were created to help produce descriptive analysis for SOS fusing rate for males and females. The results show closure rate (percentage of SOS closure/year) in females is faster than males, with fastest rate at age 10 years (38.60% ± 19.64%) and 11years (32.26% ± 11.86%), which then declines to reach less than 10% per year by age 15. The closure rates in males appear slower than females at age 10 and 11, but they last a longer time, ranging between 22–26% per year from age 11 to 14 years, then the rates decline to reach around 17% per year by age 15 years (Table 7, Table 8, Fig 6 and Fig 7).

**Table 7:** Spheno-occipital synchondrosis closure rate in males (percentage / year) between 9–16 years.

Age group	Age range	Number	Mean rate	SD	Range
9	8.5–9.4	1	7.47	–	–
10	9.5–10.4	5	14.69	10.98	2.40–30.30
11	10.5–11.4	20	23.14	11.46	5.45–46.43
12	11.5–12.4	30	24.61	15.60	3.22–67.00
13	12.5–13.4	36	26.69	11.58	3.24–44.44
14	13.5–14.4	36	22.32	14.14	2.80–54.55
15	14.5–15.4	25	17.12	13.01	2.40–46.15
16	15.5–16.4	6	15.32	13.76	4.40–46.15

**Table 8:** Spheno-occipital synchondrosis closure rate in females (percentage / year) between 9–16 years.

Age group	Age range	Number	Mean rate	SD	Range
9	8.5–9.4	4	19.53	16.09	4.94–40.00
10	9.5–10.4	16	38.60	19.64	19.67–101.4
11	10.5–11.4	36	32.26	11.86	5.60–57.14
12	11.5–12.4	24	22.95	13.46	2.88–44.16
13	12.5–13.4	22	17.82	15.80	1.55–57.14
14	13.5–14.4	11	12.04	7.25	3.72–27.31
15	14.5–15.4	6	8.04	3.75	5.33–12.20
16	15.5–16.4	2	7.62	2.28	6.00–9.23

The association between SOS closure and puberty onset in females was evaluated. Based on the review of records for females between ages 9 and 15 years, all female patients who had CBCT and answered if menarche had commenced were selected. This resulted in 175 females, who answered the question and had CBCT at the same time. The results show that there was a significant association between the SOS closure stage and whether the menstrual cycle had started (Fisher's Exact Test p < 0.001). In females, who had reached menarche, we see the lowest distribution in Stage 0 (open SOS), and we find the highest distribution in Stage 3 (complete fusion). Whenever the menarche did not start, the highest distribution was found in Stage 0 and the lowest distribution in Stage 3 (Table 9).

**Table 9:** Menarche commencement distributions among spheno-occipital synchondrosis closure stages.

	Menarche Commencement		
	Yes	No	Total
Stage 0	2(8.7%)	21(91.3%)	23 (100%)
Stage 1	16(61.5%)	10(38.5%)	26(100%)
Stage 2	32(82%)	7(18%)	39(100%)
Stage 3	84(96.5%)	3(3.5%)	87(100%)
Total	134(76.5%)	41(23.5%)	175(100%)

## Discussion

The objectives of this study were to document SOS closure pattern and timing in a large modern American sample with known-ages and to evaluate the relationship between SOS closure and puberty in females. We calculated the rate of SOS closure in males and females. This morphological and physiological information is valuable to increase our understanding of craniofacial growth and skeletal development, and it would have different applications in related clinical and anthropological fields

The results of this study show significant positive relationship between age and stage of spheno- occipital closure for both sexes (male: *r*_*s*_ = 0.844; sig = P < 0.001 female: *r*_*s*_ = 0.834; sig = P < 0.001) (Fig 4). Our results are comparable to that of Frankel et al. [17], who has male: *r*_*s*_ = 0.821 (n = 143)), who used a similar 4-Stages approach for SOS evaluation based on the assessment of CT images.

Based on mean ages in our sample (**Table 3**and Fig 5), we found complete SOS closure (Stage 3) attainment occurs at an age equal to or older than 16.41 years (± 1.40 years) and 15.25 years (± 1.89 years) in males and females, respectively. These ages are in accordance with mean ages reported in the literature [6,10,12,14,15,19,21,22,29]. Our study shows the mean age of complete SOS opening (Stage 0) are 11.07 years (± 1.91) and 9.75 years (±1.3) for males and females, respectively. These ages are consistent with those age means reported in literature [7,17–19,23,30].

One goal in descriptive studies of SOS closure is to find the mean age of youngest individuals with completely fused SOS (Stage 3) and the mean age of oldest individuals with completely open SOS (Stage 0). However, it is worth mentioning that the youngest and oldest ages are affected by sample compositions and age limits. Some studies have samples that include individual ages up to 25 years, and they reported Stage 3 closure age in both sexes around 20 years and above [18,30]. The same could be said when we calculate the mean age of oldest individuals with completely open SOS (Stage 0). Some studies have a high percentage of newborn babies in their samples and reported a mean age of Stage 0 occuring at age less than 4 years in both sexes [4,20,22]. However, sample composition and staging systems are not the only methodological factors that could contribute to age differences of SOS closure. Different scoring methodology (such as direct inspection of skeletal material, autopsy, bone histology, and computed tomography) could affect scoring accuracy and the age of closure [6,31]. Different CT and CBCT studies used different resolution, and this could be another factor that affected the accuracy of SOS scoring. The CBCT records of the current study were colleceted from orthodontic clinic; using the CBCT has an advantage over conventional CT, as it could produce good quality images with less radiation. This low radiation protocol might affect the resolution of the images, especially when scoring the very beginning stage of the fusion and the end stage. This variation in scoring based on the quality of the images is considered an inherent limitation in any scoring method that uses radiography.

Previous studies have concluded the SOS fuses earlier in girls than boys [4,12,14,15,17,20,22,32–34]. The observations of the present study corroborate the earlier studies, as each SOS closure stage consistently appeared earlier in girls than boys. The SOS Stages in the female groups are always ahead of the male groups by at least one year.

In the current study, the association between closure of SOS and puberty onset in females was evaluated. The results indicate that there was a significant association between the SOS closure stage and whether the menstrual cycle has started. By the time the menstrual cycle started, the SOS will be fused. This result could be analyzed compared to what Simmons & Gruelich (1943) suggested that menarcheal age is always preceding the skeletal age. Even though our study did not examine the menarcheal age, we found the same pattern with menarche; it precedes SOS fusion most of the time [35]. This result is in agreement with Björk & Helm [36]. Investigators have found menarche never occurred before peak height growth velocity [37,38]. In addition, the growth literature has shown a strong correlation between the peak height velocity and peak of facial growth [39,40]. In a recent study published by Demirturk Kocasarac et al. [41], they found good correlation between cervical vertebra maturation that occurs during adolescence age and SOS fusion. All these together suggest SOS closure onset may precede the menarche and might occur at the same time as highest rate of facial growth. However, a further study with more focus on the relationship between SOS closure and facial growth is suggested.

The spheno-occipital synchondrosis seems to have an important role in the ontogeny of the cranial base and the whole human skull up to adult life [8]. According to Roche et al. [42], the cranial base elongates more in boys than in girls during pubescence and adolescence. They also added that pubertal elongation occurs earlier in girls, with 95% level of adult cranial base length reached 1.6 to 3.3 years earlier in girls than boys. Our results show females reach complete SOS closure around 1.2 years earlier than boys, which is relatively in agreement with Roche and colleagues [42]. We calculated the rate of SOS closure by using longitudinal data and found closure rate in females is faster than males at age 10 and 11 years. However, SOS closure in males takes longer, which makes the rate of closure faster than females at later ages (Table 7, Table 8, Fig 6 and Fig 7). However, the rate numbers must be interpreted with caution, because it considers the time between 2 scans, which differs from the time of actual closure. We could have more accurate measures if we included cases at Stage 1 and 2 only. This would show the exact closure rate and time between T1 and T2; however, because of the sample limitations, this was not possible.

Based on regression analysis, we could use SOS status to estimate chronological or skeletal age, which has applications in the anthropological and forensic medicine fields. This study has produced reliable models for both males and females (**Table 4**). Based on this regression model, age range prediction intervals were generated for each SOS closure stage in males and females using the 68% and 95% prediction intervals (**Table 6**). Calculation of an age range for SOS closure Stages was done by separate equations for males and females. In each equation, the 95% CI values of β coefficient for each SOS stage were calculated with a constant and used to predict age range of SOS closure for each stage (**Table 5**). However, as we explained earlier, the relationship between the SOS closure and puberty and eventually the facial growth potentials, this model could have a clinical value and be used to predict the age of initial closure of SOS. From this model, we could expect to have Stage 1 at age 10.06–13.26 years and 11.34–14.54 years in females and males, respectively. These ranges represent partial closure of SOS, so the cranial base and facial growth are still active. This finding requires further examination, although we expect it will have important clinical applications in craniofacial growth assessments and orthodontic treatment.

In this study, we could conclude that there is a significant relationship between puberty and SOS closure, suggesting its closure is at least partially affected by systemic, hormonal changes in the growing adolescent. It can be suggested that SOS closure can also be a biological indicator of the onset of puberty and contributing to its use as an indicator of chronological age. If we are studying a female cadaver with unknown age, we could use SOS closure to predict her maturational age: if she has open SOS, then we could suggest the cadaver was young and less than pubertal age; if she has incomplete closure (Stage 1 or 2), we could predict she is at the puberty age, and finally, if she has fused SOS, we could predict she already passed the puberty interval. However, SOS closure should not be a single indicator for age prediction and still needs supplemental biological indicators to reach more accurate age prediction.

While Scheuer and Black [16] proposed the onset of SOS closure corresponds with sexual maturity, this study represents the first investigation of the association between spheno-occipital synchondrosis closure and female puberty. Our findings confirm the sexual dimorphism of SOS closure and showed mean ages of SOS active closure (Stage 1 and 2) are correlated with mean ages of pubertal facial growth. Also, we found a significant association between menarche commencement in females and SOS closure and calculated the rate of SOS closure, which occurs at a faster rate in females and at an earlier age compared to males.

## Supporting information

S1 Table Individual ages and scores of spheno-occipital synchondrosis. (XLSX)

S2 Table Females’ menarche commencement and spheno-occipital synchondrosis scores. (XLSX)

S1 Protocol Method of 3-D image orientation. (PDF)
