# Correction: Timing and rate of spheno-occipital synchondrosis closure and its relationship to puberty

**DOI:** 10.1371/journal.pone.0191703

**Published:** 2018-01-19

**Authors:** Anwar Alhazmi, Eduardo Vargas, J. Martin Palomo, Mark Hans, Bruce Latimer, Scott Simpson

There are two additional affiliations missing for the first author. Anwar Alhazmi should also be affiliated with: #3 Department of Anatomy, School of Medicine, Case Western Reserve University, Cleveland, Ohio, USA and Department of Orthodontics, College of Dentistry, Jazan University, Jazan, Saudi Arabia.
