# Supplementary material for: Timing and rate of spheno-occipital synchondrosis closure and its relationship to puberty
Source: PLoS One. 2017 Aug 21;12(8):e0183305. doi: 10.1371/journal.pone.0183305 (PMC5565182; doi:10.1371/journal.pone.0183305)
Supplement: S1 Protocol — (PDF) [file pone.0183305.s003.pdf]

Method of 3-D image orientation

The slice thickness was set at 0.38 mm as a standard Voxel size. When the bilateral nerve canals were not simultaneously visible on one slice, the slice thickness was increased until they were. The method followed in this study was described by Wu et al(1) in 2011 to orient each one of the pre- and post-treatment 3-D images: In the coronal view, the axial plane was aligned with the center of the left and right optical foramina by tilting the head to avoid head roll, (Figure1). In the axial view the coronal plane was aligned with the center of the left and right foramina ovale by rotating the head to avoid head yaw and then the axial plane was scrolled to the level of Foramen cecum. The sagittal plane was positioned to pass through Foramen cecum. (Figure2). Finally, in the sagittal view, the pitch of the head was adjusted so that the axial plane overlapped McRae's line (Basion to Opisthion) (Figure 3). This process allowed the image to be fixed in the 3 planes of space and without rotating the image in any direction; the axial plane overlapping McRae's line and the sagittal plane overlapping Foramen Cecum, the coronal plane was dragged back to Basion. The Origin, or (0,0,0) coordinate, was estimated around the Basion area because this is where all 3 planes intersect. This orientation and coordinate system was saved as default orientation.

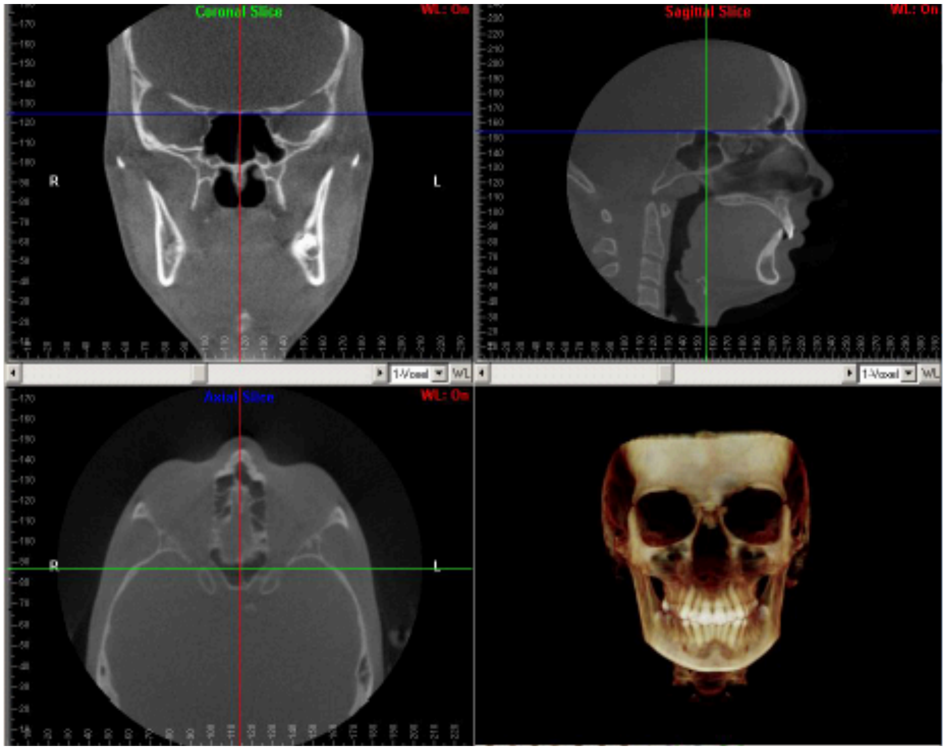

Figure 1 Axial plane setting according the Optical Foramina in coronal view.

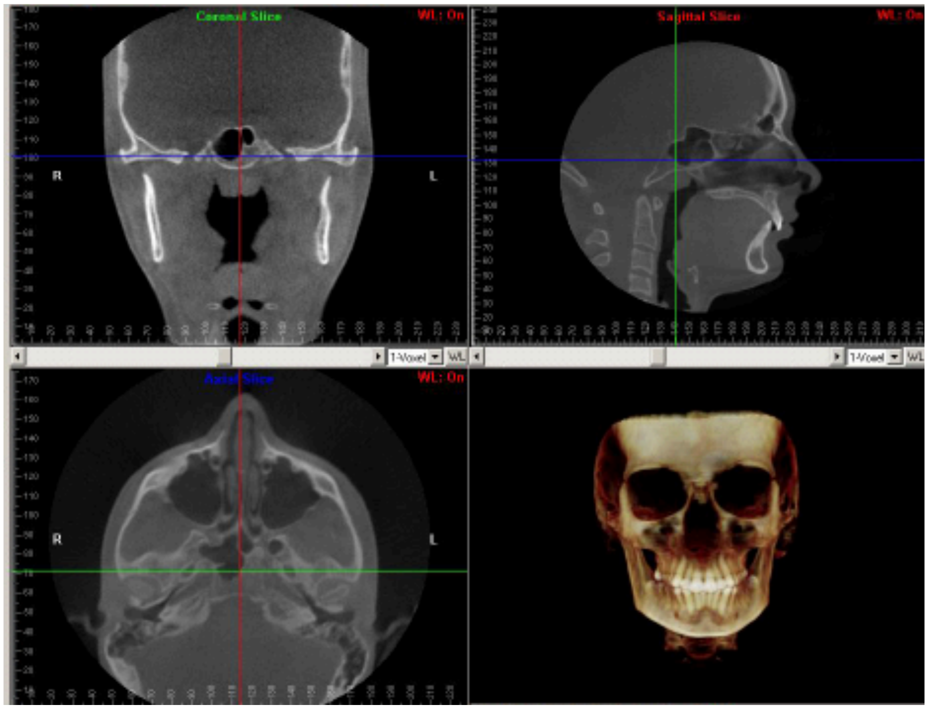

Figure 2 Coronal plane setting according to foramina ovale in the axial plane

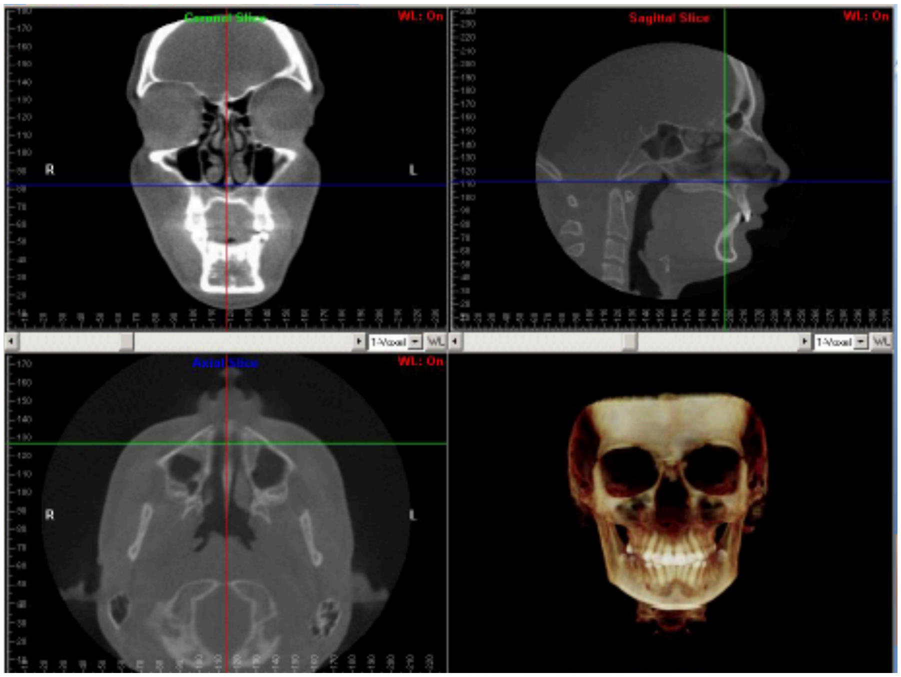

Figure 3 Axial plane setting using foramen cecum and McRae's line in the sagittal plane.

----

1. Wu R, Palomo JM, Landers M, Hans MG. Establishing a superimposition method to compare results in pre- and post-treatment with CBCT: Part I: Stable cranial-base landmarks for three dimensional superimposition of CBCT - Master's Thesis. Case Western Reserve University; 2011.
